# A Novel Unsupervised Segmentation Approach Quantifies Tumor Tissue Populations Using Multiparametric MRI: First Results with Histological Validation

**DOI:** 10.1007/s11307-016-1009-y

**Published:** 2016-10-12

**Authors:** Prateek Katiyar, Mathew R. Divine, Ursula Kohlhofer, Leticia Quintanilla-Martinez, Bernhard Schölkopf, Bernd J. Pichler, Jonathan A. Disselhorst

**Affiliations:** 1 Werner Siemens Imaging Center, Department of Preclinical Imaging and Radiopharmacy, Eberhard Karls University Tuebingen, Roentgenweg 13, 72076 Tuebingen, Germany; 2 Max Planck Institute for Intelligent Systems, Tuebingen, Germany; 3 Institute of Pathology and Neuropathology, Eberhard Karls University Tuebingen and Comprehensive Cancer Center, University Hospital Tuebingen, Tuebingen, Germany

**Keywords:** Tumor heterogeneity, Multiparametric MRI, Spectral clustering, *K*-means, Fuzzy C-means, Gaussian mixture modeling

## Abstract

**Purpose:**

We aimed to precisely estimate intra-tumoral heterogeneity using spatially regularized spectral clustering (SRSC) on multiparametric MRI data and compare the efficacy of SRSC with the previously reported segmentation techniques in MRI studies.

**Procedures:**

Six NMRI nu/nu mice bearing subcutaneous human glioblastoma U87 MG tumors were scanned using a dedicated small animal 7T magnetic resonance imaging (MRI) scanner. The data consisted of T2 weighted images, apparent diffusion coefficient maps, and pre- and post-contrast T2 and T2* maps. Following each scan, the tumors were excised into 2–3-mm thin slices parallel to the axial field of view and processed for histological staining. The MRI data were segmented using SRSC, *K*-means, fuzzy C-means, and Gaussian mixture modeling to estimate the fractional population of necrotic, peri-necrotic, and viable regions and validated with the fractional population obtained from histology.

**Results:**

While the aforementioned methods overestimated peri-necrotic and underestimated viable fractions, SRSC accurately predicted the fractional population of all three tumor tissue types and exhibited strong correlations (r_necrotic_ = 0.92, r_peri-necrotic_ = 0.82 and r_viable_ = 0.98) with the histology.

**Conclusions:**

The precise identification of necrotic, peri-necrotic and viable areas using SRSC may greatly assist in cancer treatment planning and add a new dimension to MRI-guided tumor biopsy procedures.

**Electronic supplementary material:**

The online version of this article (doi:10.1007/s11307-016-1009-y) contains supplementary material, which is available to authorized users.

## Introduction

Targeted cancer therapies have experienced an unprecedented increase in approval over the past decade [1], with most recent approaches utilizing the immune system against tumors. However, due to their cytostatic effects, in treatment response evaluation of these therapies, volume- and size-based descriptors (WHO and RECIST criteria) need to be complemented with quantitative imaging biomarkers [2].

A plethora of studies have reported the prognostic value of the multiparametric magnetic resonance imaging (MRI) derived quantitative biomarkers in oncology [3, 4]. Nonetheless, little effort has been laid out in developing techniques to quantify the intra-tumoral heterogeneity. Several investigations have used *K*-means clustering [5] or related algorithms to distinguish necrosis from viable tissue and assess phenotypic variability [6–9]. Kazerooni et al. [10] combined fuzzy C-means (FCM) with a region growing algorithm to segment glioblastoma in patients. In addition to these, a recent study [11] has demonstrated the application of Gaussian mixture modeling (GMM) [5] on longitudinal positron emission tomography (PET)/MRI data to create a spatio-temporal profile of different tumor tissue populations.

All of the previously mentioned techniques make strong assumptions about the shape of the clusters and are classified as partitional clustering algorithms [5]. These methods perform well as long as the clusters are easily separable and their underlying assumptions are met. However, due to highly composite microenvironment and voxel level perturbations, the multidimensional MRI tumor data may contain mixed and irregularly shaped clusters (in parameter space), limiting the applicability of the aforementioned algorithms.

In this paper, we propose a robust algorithm, which overcomes the limitations of the previously described techniques and accurately characterizes the tumor tissue variability. We show that spatially regularized spectral clustering (SRSC) outperforms *K*-means, FCM, and GMM. Furthermore, we quantitatively validate the segmentation results of SRSC on the MRI data (consisting of apparent diffusion coefficient (ADC) maps, normal and contrast-enhanced T2 and T2* maps) using tumor histology.

## Materials and Methods

### Data Acquisition

All the studies were performed in accordance with the German Animal Welfare Act and protocols were approved by the Regierungspraesidium in Tuebingen. Human U87MG glioblastoma tumor cells were subcutaneously inoculated in the right shoulder of six 11-week-old NMRI/nu-nu mice (1 × 10^7^ in 200 μl of 0.9 % NaCl). Once injected, the tumors were allowed to grow for 2 weeks, after which the imaging experiments were carried out.

The MRI scans were acquired using a dedicated small animal 7T ClinScan scanner (Bruker BioSpin, Ettlingen, Germany). The details of the MRI sequences used for the acquisition of T2-weighted anatomy, ADC, T2 and T2* images are provided in the supplementary material. The pre- and post-contrast T2 and T2* images were obtained before and 2 min after the intravenous injection of 50 μl of ferumoxytol (Rienso; Takeda Pharmaceuticals, Glattpark-Opfikon, Switzerland). To avoid motion artifacts, the animal breathing was tracked (Model 1030; SA Instruments, Stony Brook, NY, USA) and used for triggering the anatomy and ADC sequences. Inveon Research Workplace (Siemens, Knoxville, Tennessee, USA) was utilized to delineate the tumors on the anatomical images of the mice.

Although not included in this paper, during MRI scans, the mice were also injected with 2-deoxy-2-[^18^F]fluoro-d-glucose PET tracer for independent investigations.

### Histology

At the end of each scan, the mice were taken out from the MRI scanner and sacrificed by cervical dislocation, while maintaining their position on the bed. Prior to dissection, three equidistant lines (∼2–4 mm apart) were drawn on the skin parallel to the imaging field of view and the tumors were frozen using a freezing spray. The frozen tumors were subsequently cut into four pieces along the parallel lines, and the sectioned parts were placed into the tissue biopsy baskets while keeping track of the slice orientation. Thereafter, the tissue baskets were placed in 4.7 % neutral-buffered formaldehyde for 48 h and processed for paraffin embedding and subsequent cutting in 6-μm sections. An automated immunostainer (Ventana Medical Systems, Tucson, AZ, USA) was used to perform the immunohistochemistry with the following primary antibodies: GLUT-1 (Glucose transporter 1, Abcam Inc., Suite B2304 Cambridge, USA), Ki-67 (Clone SP6, DCS Innovative Diagnostik-Systeme GmbH u. Co. KG, Hamburg, Germany), and CD-31 (Abcam plc, 330 Cambridge Science Park, Cambridge, UK). Positive and negative controls were included for the immunohistochemical analysis of each antibody. Additionally, H&E staining was performed. The stained histology slides were scanned into high-resolution digital images using a NanoZoomer 2.0 HT (Hamamatsu, Hamamatsu City, Japan), and different tumor tissue populations were marked by a seasoned mouse pathologist. Utilizing these markings as reference, regions of interest were drawn on the histology slices using NDP.view (Hamamatsu, Hamamatsu City, Japan) and the fractional population of each tumor tissue type was calculated. The viable and necrotic tissues were delineated on H&E, while the peri-necrotic tissue was defined on GLUT-1 immunostaining. The registration (additional details are provided in the supplemental data) between histology and delineated tumor images was performed manually using MATLAB (MathWorks, Natick, MA, USA), as described by Divine et al. [11]. Due to inadequate registration, one mouse was excluded from further analyses.

### Spatially Regularized Spectral Clustering

Spectral clustering [12, 13] utilizes voxel-wise MRI feature vectors to create affinity matrix for each tumor. The voxel-wise feature vectors were obtained by concatenating the co-registered MRI parameters (ADC, T2 pre-contrast, T2 post-contrast, T2* pre-contrast, and T2* post-contrast), and the affinity matrices were constructed using a radial basis function (RBF) kernel:

$$ {W}_{ij}=\left\{\begin{array}{cc}\hfill {e}^{-\left\Vert {x}_i-{x}_j\right\Vert {}^2/2{\sigma}^2}\hfill & \hfill \mathrm{if}\ i\ne j\hfill \\ {}\hfill 0\hfill & \hfill \mathrm{otherwise}.\hfill \end{array}\right. $$

Here, *σ* is the scale parameter of the RBF kernel and ‖*x*_*i*_ − *x*_*j*_‖ is the pairwise Euclidian distance between the MRI feature vectors of voxel *i* and *j*. Unsupervised clustering was performed using constrained GMM on the eigenvectors of the normalized Laplacian matrix. Further details about the algorithm can be found in the supplemental data.

Standard GMM probabilistically assigns observations to different clusters and characterizes them using a mean vector and covariance matrix. We included a spatial regularization (in the image space) into standard GMM which we will refer to as constrained GMM. Spatial constraints were imposed by weighing the tissue class probabilities of each voxel by the average tissue class probabilities of the 26 connected neighboring voxels during the optimization process. Thus, the likelihood of a voxel to be characterized as a certain tissue type is enhanced if the nearby voxels belong to the same tissue class and vice versa.

The results of SRSC were compared with *K*-means, FCM, and standard GMM. All of the methods were implemented in MATLAB.

### Statistical Analysis

The Pearson’s correlation coefficient was computed to evaluate the linear relationship between the histology and clustering tumor tissue fractions. The one-sample Kolmogorov-Smirnov test was used to test whether the distribution was normal. Due to non-normality, the differences between all of the groups were first checked using the Kruskal-Wallis non-parametric test. In case of a significant difference, the individual groups were compared using the Bonferroni corrected rank-sum tests (a *p* value less than 0.0167 was considered as statistically significant).

## Results

**Fig. 1.**
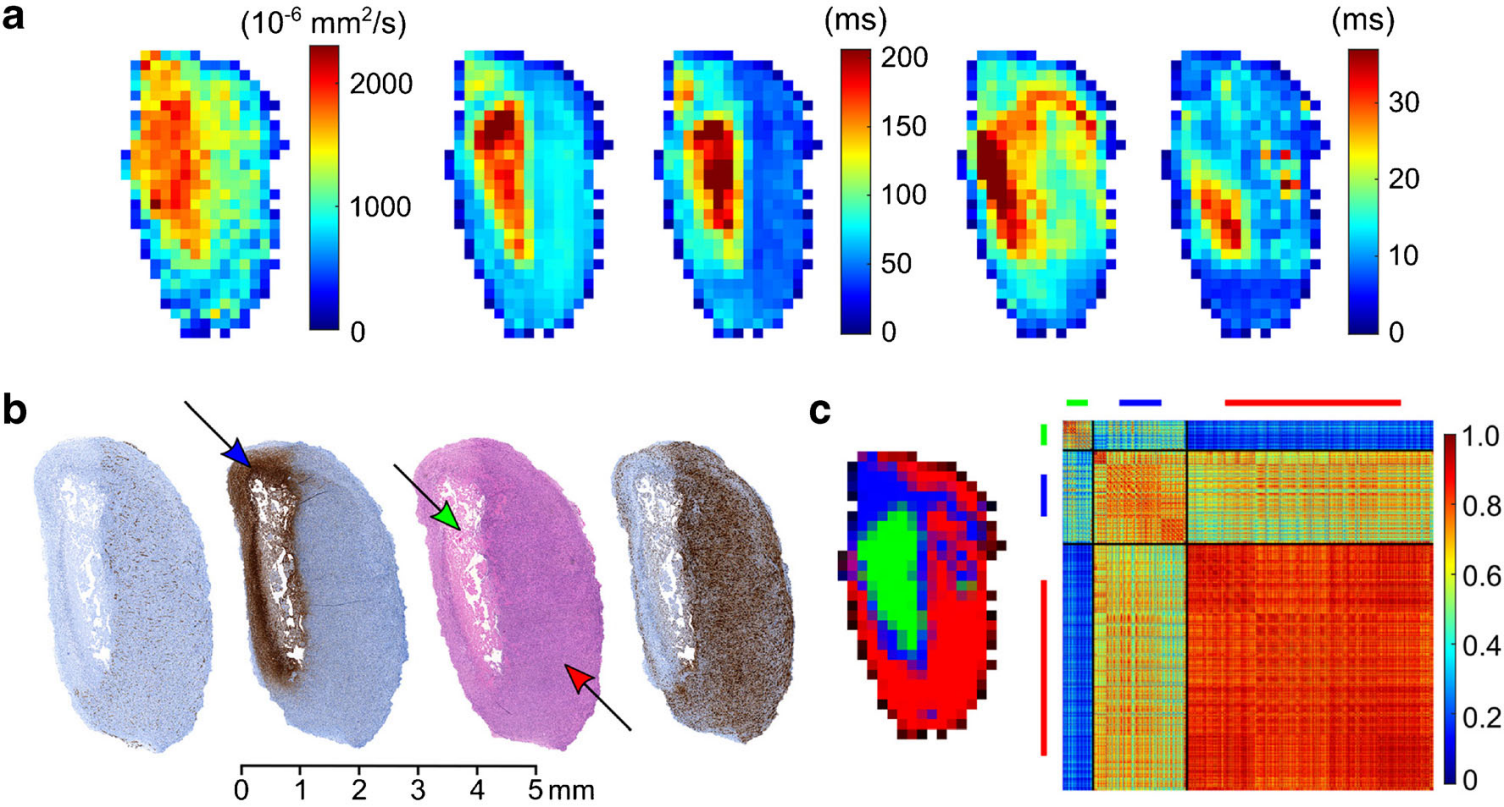
**a** The acquired MRI parameters of a tumor, left to right: ADC, T2 pre, T2 post, T2* pre, and T2* post maps. **b** Left to right: CD-31, GLUT-1, H&E, and Ki-67 stained histology of the tumor from **a**. **c** Left to right: SRSC probability map and the affinity matrix of the tumor. *Green*, *blue*, and *red colors* represent the necrotic, peri-necrotic, and viable tissue, respectively. The *arrows* in the histology indicate the corresponding tissue type in the tumor. The affinity matrix was computed using the voxel-wise feature vectors from the entire tumor volume. The *diagonal* and *off-diagonal matrices* in the affinity matrix depict intra- and inter-class similarities for the labeled tissue clusters, respectively. For example, the high intra-class similarity of viable cluster indicates the presence of homogeneous viable areas in the tumor.

The MRI parameters of one of the tumors are shown in Fig. 1a. The corresponding histology and segmentation results of SRSC are presented in Fig. 1b, c, respectively. The affinity matrix (Fig. 1c) depicts the intra- and inter-class similarity between the identified tissue classes of the tumor.

**Fig. 2.**
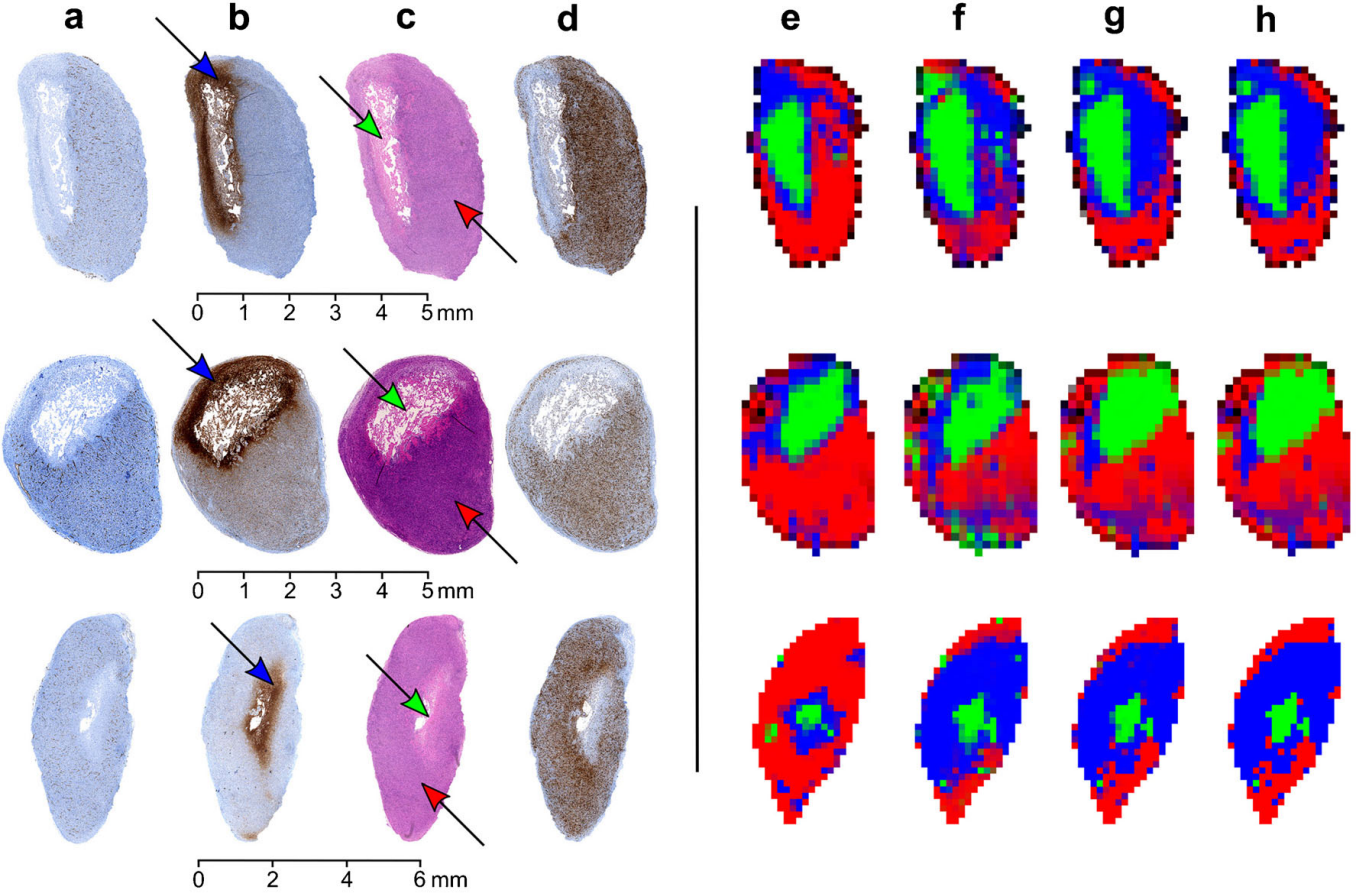
**a** CD-31, **b** GLUT-1, **c** H&E, and **d** Ki-67 stained histology of three different tumors and corresponding segmentation maps obtained using **e** SRSC, **f** GMM, **g** FCM, and **h** K-means. *Green*, *blue*, and *red colors* represent the necrotic, peri-necrotic, and viable tissue, respectively. The *arrows* in the histology indicate the corresponding tissue type in the tumor.

The clustering comparisons of SRSC with *K*-means, FCM, and standard GMM, together with the histological images of three tumors are shown in Fig. 2. The proposed method outperformed all three techniques and demonstrated the best visual correlation with the histology. The SRSC results of the remaining tumors are shown in Supplementary Figs. 1 and 2. Both tumors were highly homogeneous and mostly composed of viable portions. SRSC however also identified minor amounts of muscle and connective tissue.

**Fig. 3.**
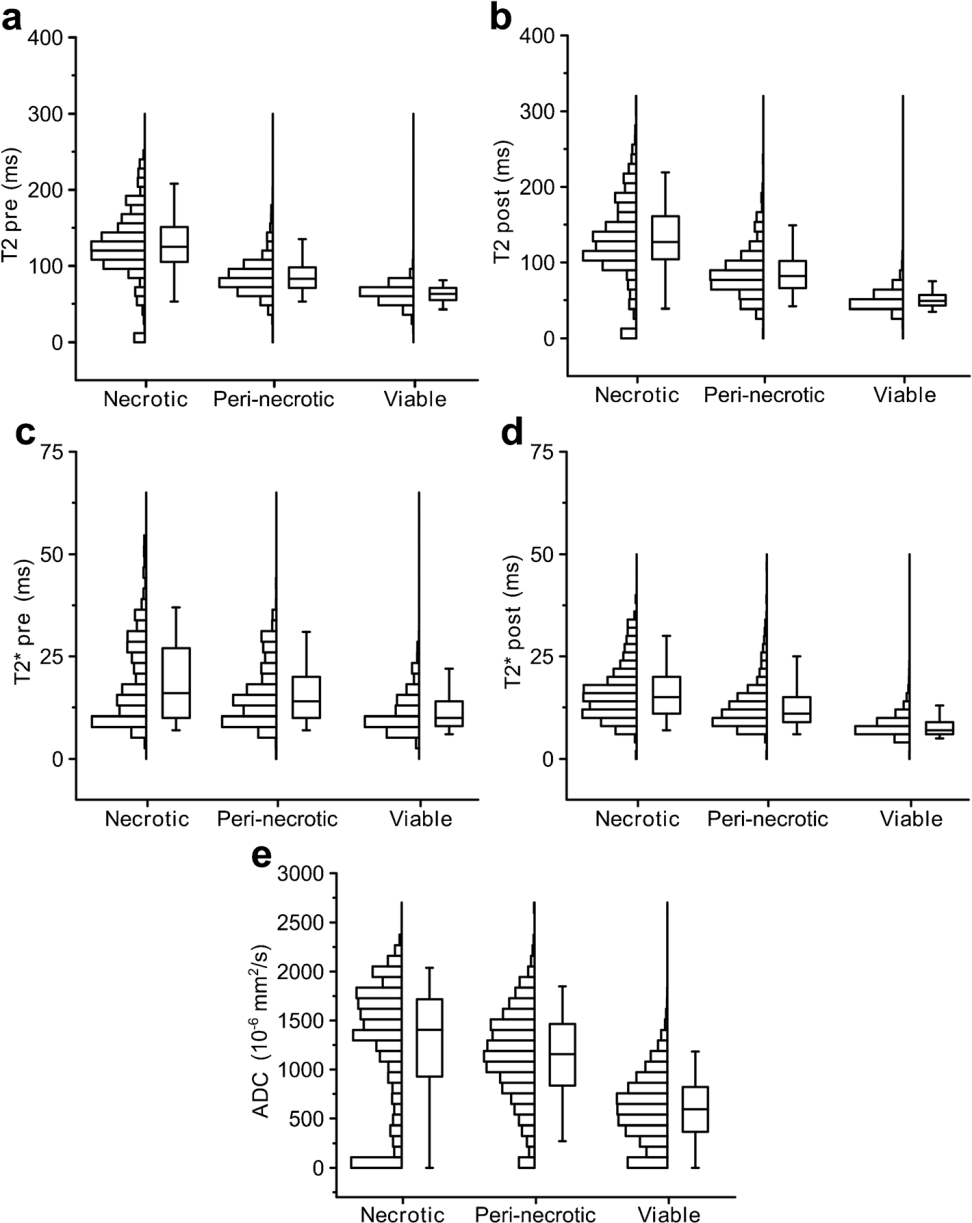
The distributions of the acquired MRI parameters for each tumor tissue type. The *boxes* depict the interquartile range and the *whiskers* extend to the 5th and 95th percent value of the parameter. The *line* in the box shows the median of the distribution.

The class-wise box plots and the histograms of all the MRI parameters for the aforementioned tumors are shown in Fig. 3. The box plots and histograms were generated using the voxel-wise segmentation results of SRSC. For each MRI parameter, the distributions of all three tumor tissue classes significantly differed from each other (Supplementary Table 1).

Table 1 summarizes the Pearson’s correlation coefficients (*p* values are given in Supplementary Table 2) between histology and clustering tumor tissue fractions for all four algorithms. Matching scatter plots are shown in Supplementary Fig. 3. While *K*-means, FCM, and GMM overestimated peri-necrotic and underestimated viable fractions, SRSC accurately predicted the fractional population of all the three tumor tissue types.

**Table 1:** Pearson’s correlation coefficients for the tissue fractions obtained from histology and clustering algorithms

Tissue type	Pearson’s correlation coefficient (r)			
	SRSC	*K*-means	FCM	GMM
Viable	0.98	0.66	0.33	0.59
Necrotic	0.92	0.76	0.79	0.88
Peri-necrotic	0.82	−0.84	−0.80	−0.72
All	0.99	0.64	0.69	0.68

## Discussion

We proposed a novel algorithm for the analysis of multi-parametric MRI data and assessment of the intra-tumoral heterogeneity. We compared our algorithm with the previously reported segmentation methods in MRI studies [6–8, 11] and exhibited its efficacy over *K*-means, FCM, and standard GMM. We corroborated the segmentation results of SRSC with different histological stainings and demonstrated strong correlations between the tissue fractions derived from immunohistochemistry and SRSC. The precise identification of the necrotic, peri-necrotic, and viable tissue fractions using SRSC highlights the strengths of combining novel image analysis methods with multiparametric imaging and advocates the potential of the proposed method for clinical investigations.

Different types of cell death play an important role in tumor regression and progression. Among others, necrosis is designated as a lethal form of cell demise, which triggers inflammation [14]. Moreover, inflammation is a known regulator of the hallmarks of cancer [15], whose complex interplay promotes uncontrolled tumor growth. Thus, measuring the amount of necrosis can be pivotal for predicting the degree of tumor aggressiveness and cancer morbidity [16]. While all of the models were able to identify necrotic regions, only SRSC provided precise estimates of viable (revealed by high mitotic rate in Ki-67 staining) and peri-necrotic tissue populations. Furthermore, the average ADC in SRSC segmented necrotic (1252.68 ± 628.48), peri-necrotic (1132.2 ± 466.72), and viable (598.46 ± 344.67) regions was also consistent with previous findings [7, 11]. The moderate and relatively low interclass similarity of the peri-necrotic tissue with the viable and necrotic regions respectively, shown in Fig. 1c (affinity matrix), might be a result of metabolic stress, elucidating the gradual transformation of the peri-necrotic regions from viable to necrotic tissue. This is also indicated by the higher expression of GLUT-1 receptor in the peri-necrotic areas in Fig. 1b, possibly due to induced hypoxia [7]. Although we did not perform any hypoxia-specific staining, it is well established that hypoxia leads to an increase in glycolysis, eventually resulting into a higher GLUT-1 expression [17]. Similar characteristics are exemplified in Supplementary Fig. 4.

In this study, the histology was manually co-registered with the *in vivo* MRI images. The confounding challenges faced in the registration process [18] were mitigated by the careful sectioning and fixation of the tumor. Due to the difficulties encountered in one-to-one (histology to imaging) registration, tissue labels from histology are hard to obtain, limiting the application of supervised algorithms. Recently, one investigation has improved this by using a two-step registration process, involving digital photographs of the specimen and later performing a linear discriminant analysis on the multiparametric MRI data [19]. Another way to circumvent the registration challenges is by using the unsupervised and supervised techniques in a cascaded manner. Specifically, tissue labels can be obtained by clustering the multiparametric MRI data using SRSC and labeled voxels with a probabilistic confidence score can be used to train a supervised classifier, thereby allowing the development of phenotype specific mathematical models.

Biopsies are routinely used in modern cancer diagnostics and tumor phenotyping. As tumors exhibit frequent spatial and temporal heterogeneity, the limited spatial extent of the invasive procedure can severely underestimate the disease complexity, resulting in a misleading prognosis or an unsuccessful therapy [20]. Imaging techniques on the other hand provide a complete view of the patient, allowing a comprehensive inspection of the spatio-temporal variations. Therefore, the combination of imaging diagnostics with tissue biopsy procedures could not only assist in lesion localization and selective tissue sampling, but could also deliver an extensive phenotypic and genotypic profile of the tumor, potentially uncovering the causal relationships between the two [21].

Since the acquired MRI parameters (ADC, T2 and T2* maps) in this investigation are standard protocols in the clinic, SRSC can be translated into clinical examinations. One major limitation of this study, however, is the use of a single xenograft tumor model and the small sample size. Evaluating SRSC on several tumor types along with a combination of different cancer therapies and imaging parameters could reveal the versatility of the suggested method and bring additional insights about the most robust and informative *in vivo* imaging biomarkers.

In multifaceted tumor microenvironment, it is highly probable that the neighboring cells exhibit similar functional and anatomical characteristics and there is a smooth transition from one tissue type to another. MRI measurements, however, can be corrupted by subject motion and magnetic field in-homogeneities, giving rise to voxel level uncertainties. We addressed these textural irregularities by imposing spatial constraints and achieved accurate intra-tumor segmentation results. As opposed to commonly used partitional clustering algorithms, SRSC makes no *a priori* assumptions about cluster shapes; hence, it is likely to perform better on multidimensional data sets. Such methods of region-wise analyses are of high significance for multi-parametric imaging, as they can facilitate biomarker selection and treatment planning by providing a reliable quantification of imaging measures probing inter- and intra-tumor heterogeneity [22].

## Conclusion

In conclusion, through quantitative histological validation and one-to-one algorithmic comparison, we demonstrated the efficacy of SRSC on multiparametric MRI data and delivered an accurate segmentation of the intra-tumoral heterogeneity. Multiparametric imaging in combination with image analysis tools has the ability to probe tumor heterogeneity beyond currently utilized volume- and size-based measures, which might be of great value for selective treatment planning and reliable response evaluation of personalized cancer therapies.

## Electronic supplementary material

ESM 1 (PDF 975 kb)
