# Supplementary material for: A Novel Unsupervised Segmentation Approach Quantifies Tumor Tissue Populations Using Multiparametric MRI: First Results with Histological Validation
Source: Mol Imaging Biol. 2016 Oct 12;19(3):391–7. doi: 10.1007/s11307-016-1009-y (PMC5332060; doi:10.1007/s11307-016-1009-y)
Supplement: Supplementary file 1 — (PDF 975 kb) [file 11307_2016_1009_MOESM1_ESM.pdf]

Electronic Supplementary Material

A novel unsupervised segmentation approach quantifies tumor tissue populations using multiparametric MRI: first results with histological validation

Journal: Molecular Imaging and Biology

Prateek Katiyar[1, 2], Mathew R. Divine[1], Ursula Kohlhofer[3], Leticia Quintanilla-Martinez[3], Bernhard Schölkopf[2], Bernd J. Pichler[1], Jonathan A. Disselhorst[1]

1: Werner Siemens Imaging Center, Department of Preclinical Imaging and Radiopharmacy, Eberhard Karls University Tuebingen, Tuebingen, Germany

2: Max Planck Institute for Intelligent Systems, Tuebingen, Germany

3: Institute of Pathology and Neuropathology, Eberhard Karls University Tuebingen and Comprehensive Cancer Center, University Hospital Tuebingen, Germany

Corresponding author:
Prateek Katiyar, M.Sc.
Eberhard Karls University Tuebingen
Department for Preclinical Imaging and Radiopharmacy
Werner Siemens Imaging Center
Roentgenweg 13, 72076
Tuebingen, Germany
Telephone: +49 7071 29 83639
Fax: +49 7071 29 4451
e-mail: prateek.katiyar@med.uni-tuebingen.de

Supplementary Materials and Methods

Animal Handling

Six-week-old NMRI nu/nu mice were ordered and allowed to acclimatize in the on-site animal vivarium before subcutaneous injection of tumor cells. Mice were kept in a specific pathogen free (SPF) environment in individually ventilated cages of type 2 long with wood chip bedding. The climate was regulated so that a temperature of 20 ± 1° C with a relative humidity of 50 ± 10% was maintained. The lighting was set at a 12 h night and day lighting scheme, and the mice were provided with food and water *ad libitum*. Tumor sizes as well as normal social activity of animals were monitored until tumors were palpable and showed signs of vascularization with a minimum length of 5 mm, before beginning the imaging experiments.

Before and during tumor inoculation and imaging experiments, mice spontaneously respired 1.5% isoflurane dissolved in 100% O_2 at a flow rate of 0.8 l/min in order to maintain a deep anesthesia. For injection of MRI contrast agent, catheters were placed in the tail vein of mice while they were under anesthesia using a 27 gauge micro lance needles. All experiments were carried out in a SPF environment [1].

Data Acquisition

The following settings were used for the T2 weighted turbo spin echo sequence: repetition time (TR) = 3000 ms, echo time (TE) = 205 ms, echo train length = 161, image size = 256 x 160 and voxel size (mm^3) = 0.22 x 0.22 x 0.22. The diffusion weighted images were acquired using a half-Fourier acquisition single-shot turbo spin echo (HASTE) sequence with five b-values (200, 400, 600, 800 and 1000 s/mm^2). Furthermore, the HASTE sequence had following settings: TR = 5000 ms, TE = 112 ms, echo train length = 256, number of averages = 4, image size = 192 x 120 and voxel size (mm^3) = 0.21 x 0.21 x 1.00. For T2 maps, the data were acquired using a spin echo sequence with 12 echo times (TE = 10, 20, 30, 40, 50, 60, 70, 80, 90, 100, 110 and 120 ms) and following parameter values: TR = 2500 ms, number of averages = 2, image size = 192 x 120 and voxel size (mm^3) = 0.21 x 0.21 x 1.00. Similarly, the data for T2* maps were acquired using a gradient echo sequence with 10 different echo times varying from 3 to 26.85 ms with an interval of 2.65 ms. The remaining parameter values were as follows: TR = 2000 ms, number of averages = 2, image size = 192 x 120 and voxel size (mm^3) = 0.21 x 0.21 x 1.00. All maps were calculated with in-house developed software in MATLAB (R2013a) using linear regression on the natural logarithm transformed imaging data. The T2 maps were calculated without the first echo (i.e., 11 echo's in total), whereas the T2* maps were calculated with all 10 echo's.

Histology

A rigid co-registration was performed between the histology and T2W images of the tumors. This was done by selecting the appropriate imaging slice and manually rotating it to match (based on visual correspondence) the corresponding histology section. The visual correspondence was based mainly on the contours of the imaging and histology slices, as little can be inferred about the tumor microenvironment using just the T2W images. Moreover, the co-registration was performed before the clustering analyses were carried out and therefore was not influenced by the segmentation results.

Furthermore, not every histology slice from each tumor could be matched adequately to imaging planes within the respective tumor. This was either due to the small size of the histology or the amount of tumor in the histology section, which corresponded to even fewer imaging voxels, reducing the ability to accurately align the contours. Thus, although clustering was performed on the imaging data from the entire tumor, the imaging to histology correlation analysis was restricted only to the tissue fractions obtained from the co-registered slices. This was also reasonable because the heterogeneity in the co-registered histology sections was not representative of the entire tumor volume.

Spatially Regularized Spectral Clustering

The normalized Laplacian was computed using the following expression:

$$L = D_{inv} * W_{ij} * D_{inv}.$$

Where, $D_{inv} = D^{-0.5}$ and D is the diagonal matrix with diagonal vector $d_i = \sum_{j=1}^{n} W_{ij}$. The parameter n is equal to the total number of voxels. The number of clusters was chosen based on the visual inspection of the affinity matrix constructed after clustering (i.e. number of block diagonal matrices visible in the affinity matrix). The algorithm was robust to the choice of the scale parameter σ and any value between 20 and 500 yielded the results as shown in Figure 2. The number of eigenvectors segmented using constrained GMM was always equal to the number of clusters. A window size of 3 x 3 x 3 was used to perform the spatial regularization. Due to small size of the tumors, we remained conservative with the selection of window size and did not attempt to optimize it. A total number of 100 iterations were used for the expectation and maximization (EM) step in constrained GMM.

K-means, Standard GMM and FCM

The number of clusters for *K*-means, standard GMM and FCM were kept the same as chosen for SRSC. *K*-means clustering was repeated 50 times, each time with a random seed point and a solution with the lowest within-cluster sum of points-to-centroid distance was selected. Standard GMM was initialized using the clustering solution obtained from *K*-means. An exponent of 1.1 was chosen for the fuzzy partition matrix in FCM. This was obtained by performing a grid search and minimizing the difference between the histology and FCM tissue fractions.

References

1. Kilkenny C, Browne WJ, Cuthill IC, Emerson M, Altman DG (2010) Improving bioscience research reporting: the ARRIVE guidelines for reporting animal research. *PLoS Biol*, *8*(6), e1000412.

Supplementary Tables

SUPPLEMENTARY TABLE 1. P-values obtained from different group comparisons made for each of the MRI parameter.

MRI parameter	Group comparison p-value*		
	Viable-Peri-necrotic	Viable-Necrotic	Peri-necrotic-Necrotic
**ADC**	<0.001	<0.001	<0.001
**T2 pre-contrast**	<0.001	<0.001	<0.001
**T2 post-contrast**	<0.001	<0.001	<0.001
**T2* pre-contrast**	<0.001	<0.001	<0.001
**T2* post-contrast**	<0.001	<0.001	<0.001

* p<0.0167 was considered statistically significant

SUPPLEMENTARY TABLE 2. P-values of Pearson's correlation coefficient for the tissue fractions obtained from histology and clustering algorithms.

Tissue type	Pearson's correlation coefficient p-value*			
	SRSC	*K*-means	FCM	GMM
**Viable**	<0.001	0.104	0.464	0.159
**Necrotic**	0.026	0.135	0.108	0.048
**Peri-necrotic**	0.090	0.074	0.104	0.169
**All**	<0.001	0.006	0.002	0.003

*p<0.05 was considered statistically significant

Supplementary Figures

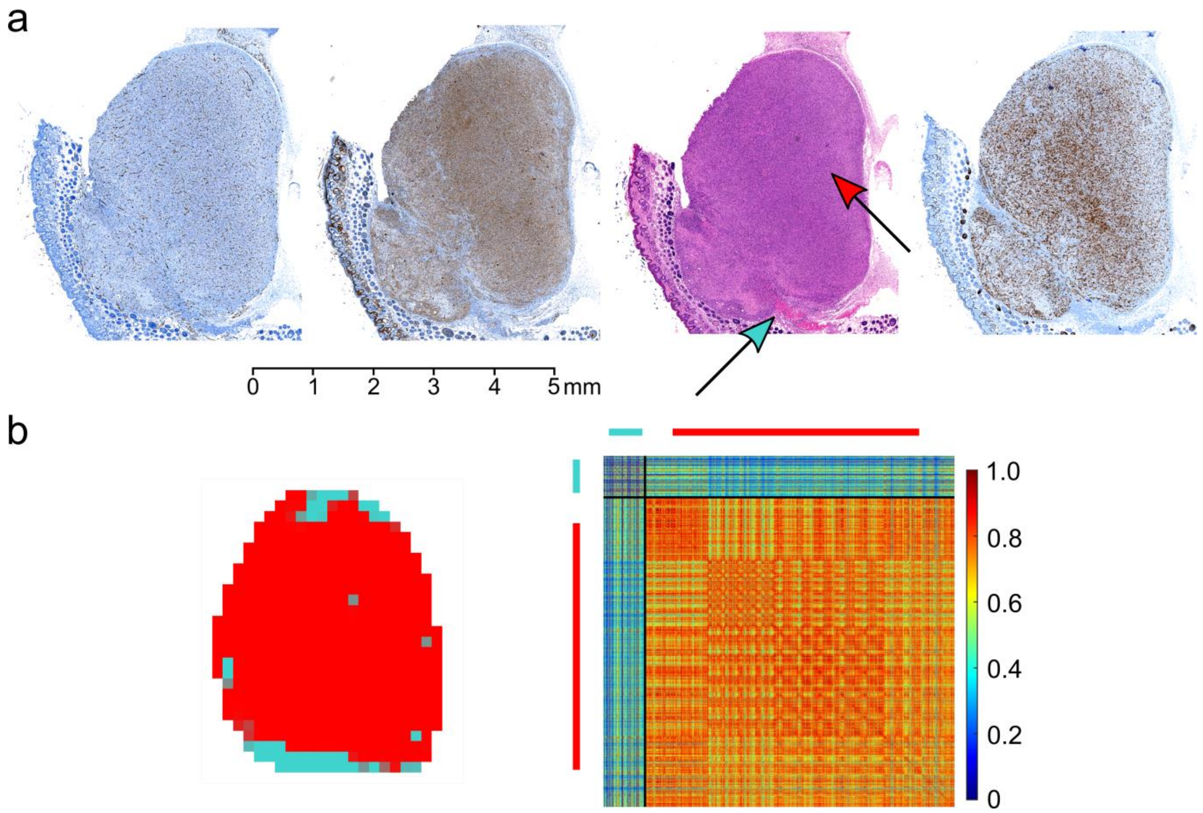

SUPPLEMENTARY FIGURE 1. (a) Left to right: CD-31, GLUT-1, H&E, and Ki-67 stained histology of tumor 4. (b) Left to right: SRSC probability map and the affinity matrix of the tumor. Red and cyan colors represent the viable, and muscle and connective tissue, respectively. The arrows in the histology indicate the corresponding tissue type in the tumor. The affinity matrix was computed using the voxel-wise feature vectors from the entire tumor volume.

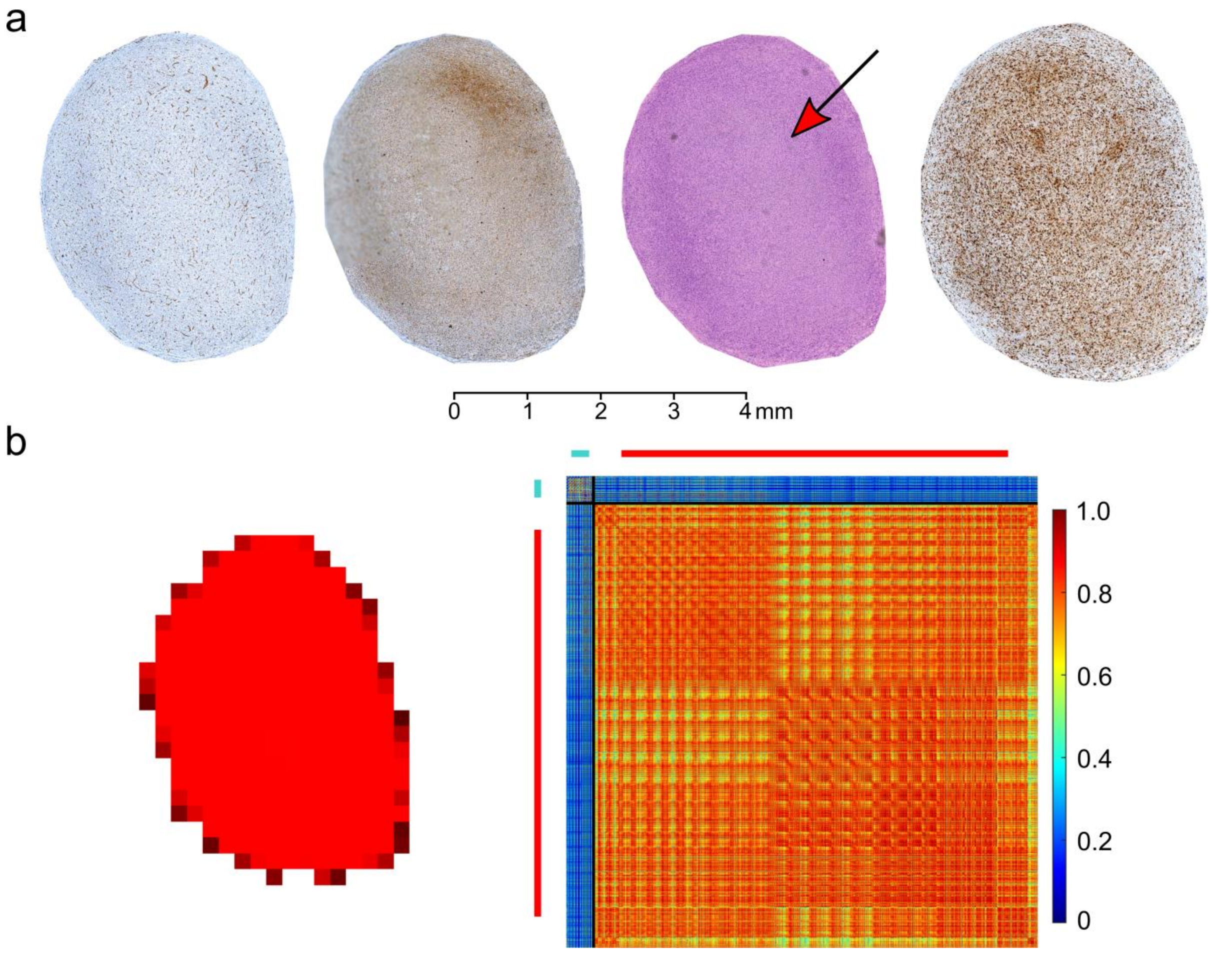

SUPPLEMENTARY FIGURE 2. (a) Left to right: CD-31, GLUT-1, H&E, and Ki-67 stained histology of tumor 5. (b) Left to right: SRSC probability map and the affinity matrix of the tumor. Red color represents the viable tissue. The arrow in the histology indicates the corresponding tissue type in the tumor. The affinity matrix was computed using the voxel-wise feature vectors from the entire tumor volume.

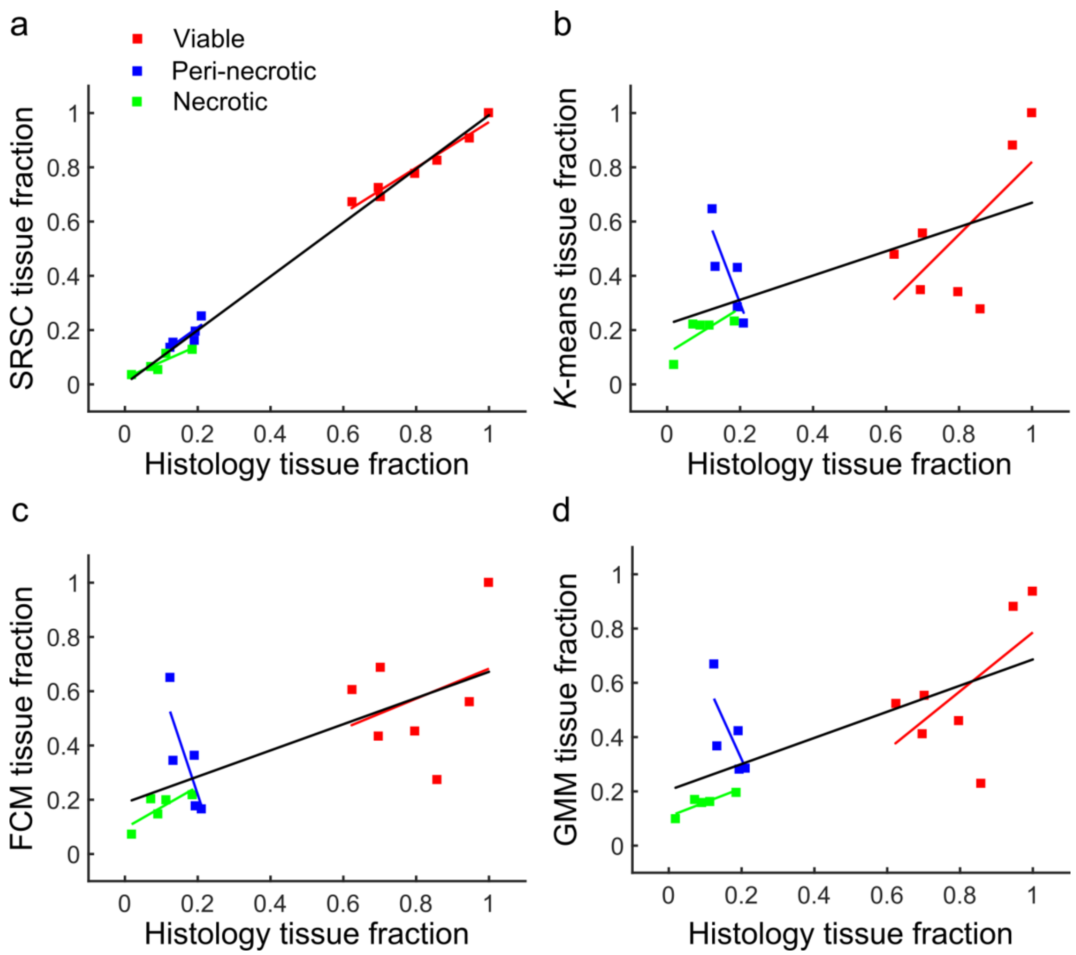

SUPPLEMENTARY FIGURE 3. Correlation plots between the histology and clustering tumor tissue fractions obtained using (a) SRSC, (b) K-means, (c) FCM and (d) GMM. The green, blue and red lines depict the linear fit on the necrotic, peri-necrotic and viable tissue fractions. The line in black shows the linear fit on the combined points from all three tumor tissue types. The five points for each tissue type were obtained from the five imaging slices matched with the respective histology of the three tumors presented in Figure 2. The two additional points in the viable group were acquired from the tumors shown in Supplementary figure 1 and 2.

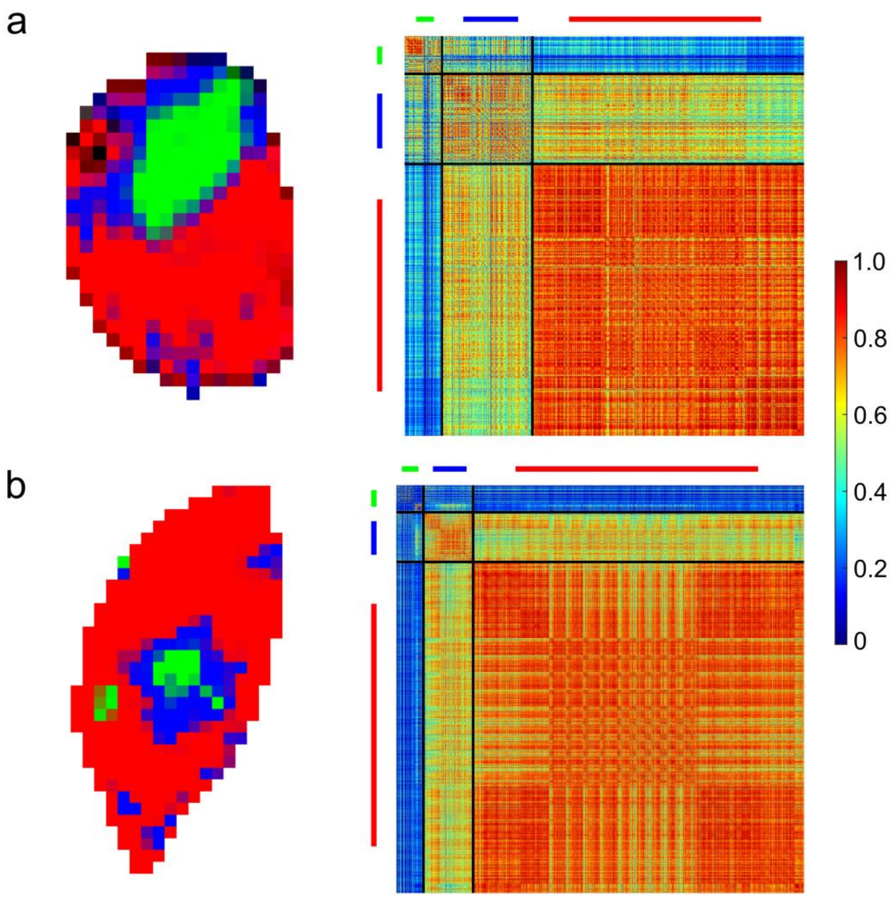

SUPPLEMENTARY FIGURE 4. Left to right: SRSC probability map and the affinity matrix of tumor (a) 2 and (b) 3. The affinity matrices were computed using the voxel-wise feature vectors from the entire tumor volume.
